# Use of Antimalarial Agents Is Associated with Favourable Physical Functioning in Patients with Systemic Lupus Erythematosus

**DOI:** 10.3390/jcm9061813

**Published:** 2020-06-10

**Authors:** Alvaro Gomez, Sofia Soukka, Petter Johansson, Emil Åkerström, Sharzad Emamikia, Yvonne Enman, Katerina Chatzidionysiou, Ioannis Parodis

**Affiliations:** 1 Division of Rheumatology, Department of Medicine Solna, Karolinska Institutet, 171 76 Stockholm, Sweden; alvaro.gomez.gonzalez@ki.se (A.G.); sofia.soukka@gmail.com (S.S.); petter.johansson@stud.ki.se (P.J.); emil.akerstrom@stud.ki.se (E.Å.); sharzad.emamikia@ki.se (S.E.); yvonne.enman@ki.se (Y.E.); aikaterini.chatzidionysiou@ki.se (K.C.); 2 Rheumatology, Karolinska University Hospital, 171 76 Stockholm, Sweden

**Keywords:** systemic lupus erythematosus, health-related quality of life, antimalarial agents, treatment, patient-reported outcomes, health perceptions, medication adherence

## Abstract

Impaired health-related quality of life (HRQoL) is a major problem in patients with systemic lupus erythematosus (SLE). Antimalarial agents (AMA) are the cornerstone of SLE therapy, but data on their impact on HRQoL are scarce. We investigated this impact using baseline data from the BLISS-52 (NCT00424476) and BLISS-76 (NCT00410384) trials (*n* = 1684). HRQoL was self-reported using the Medical Outcomes Study short-form 36 (SF-36), functional assessment of chronic illness therapy (FACIT)-Fatigue and 3-level EuroQoL 5-Dimension (EQ-5D) questionnaires. Patients on AMA (*n* = 1098/1684) performed better with regard to SF-36 physical component summary, physical functioning, role physical, bodily pain, FACIT-Fatigue, EQ-5D utility index and EQ-5D visual analogue scale scores. The difference in SF-36 physical functioning (mean ± standard deviation (SD): 61.1 ± 24.9 versus 55.0 ± 26.5; *p* < 0.001) exceeded the minimal clinically important difference (≥5.0). This association remained significant after adjustment for potential confounding factors in linear regression models (standardised coefficient, β = 0.07; *p* = 0.002). Greater proportions of AMA users than non-users reported no problems in the mobility, self-care, usual activities and anxiety/depression EQ-5D dimensions. AMA use was particularly associated with favourable HRQoL in physical aspects among patients with active mucocutaneous and musculoskeletal disease, and mental aspects among patients with active renal SLE. These results provide support in motivating adherence to AMA therapy. Exploration of causality in the relationship between AMA use and favourable HRQoL in SLE has merit.

## 1. Introduction

Systemic lupus erythematosus (SLE) is a chronic inflammatory multisystem disease that commonly affects women during their reproductive life span. It is characterised by relapses and periods of remission, and permanent organ damage may accrue during the course of the disease [1]. SLE negatively affects the patients’ health-related quality of life (HRQoL), not only because it causes pain and physical dysfunction, but also because it is associated with end-organ damage, several comorbidities and medication-related adverse events [2]. Certain disease characteristics signify particular propensity for HRQoL diminutions, i.e., early disease onset, and cutaneous, musculoskeletal and renal involvement [3,4].

Treatment of SLE includes broad immunosuppressants or immunomodulatory agents, aiming for remission or low disease activity state [5,6]. Antimalarial agents (AMA) are considered the cornerstone of SLE therapy and are recommended for all patients with SLE, unless contraindicated [7]. Administration of AMA reduces the probability of disease relapses and contributes to long-term remission, reduces the rate of organ damage accrual, increases patient survival, and is associated with protective effects against complications and comorbidities, e.g., cardiovascular disease and impairment of the renal function [8,9,10].

The concept of the patients’ perspective as an integral part of the clinical evaluation gains increasing acknowledgment within the SLE researcher community, and HRQoL outcomes are nowadays commonly used in drug trials [11]. This has to be seen as a paradigm shift, knowing that the patients’ perspective historically has rather been neglected in clinical practice. However, data on the impact of AMA on HRQoL among SLE patients are scarce and conflicting, with some studies reporting beneficial effects [12,13] while other investigations show no impact [14].

Our aim in the present study was to determine the impact of AMA on self-reported physical and mental HRQoL in a large SLE population from two phase III clinical trials.

## 2. Experimental Section

### 2.1. Study Design and Population

We conducted a post-hoc analysis of data from two multicentre, double-blinded placebo-controlled phase III trials of belimumab, i.e., BLISS-52 [15] and BLISS-76 [16], which comprised 865 and 819 participants, respectively. The two trials included SLE patients of 18 years of age and above, classified having SLE according to the American College of Rheumatology revised criteria [17]. All patients in the trials were seropositive, defined as having an ANA titre of ≥1:80 and/or anti-double stranded (ds)DNA antibody level ≥ 30 IU/mL, and had an active SLE disease defined as a Safety of Estrogens in Lupus National Assessment-Systemic Lupus Erythematosus Disease Activity Index (SELENA-SLEDAI) [18] score of 6 or more.

All patients recruited were also on a stable background treatment comprising glucocorticoids, AMA and/or immunosuppressants, or, in the majority of the cases, combinations thereof (termed standard of care therapy), for at least one month prior to treatment initiation.

For the purpose of the present study, we utilised baseline data only in a cross-sectional manner, i.e., data obtained prior to exposure to belimumab or placebo. The almost identical trial designs facilitated utilisation of pooled data from both trials. Data were made available by GlaxoSmithKline (Uxbridge, UK) through the Clinical Study Data Request (CSDR) consortium.

The patients’ rights, privacy and safety were protected in compliance with the ethical principles of the Declaration of Helsinki. Written informed consent was obtained from all study participants prior to enrolment in the BLISS-52 and BLISS-76 trial programmes. The study protocols from all participating centres were reviewed and approved by regional ethics review boards, and the study protocol for this post-hoc analysis was reviewed and approved by the regional ethics review board in Stockholm, Sweden (reference number 2019-05498).

### 2.2. Evaluation of HRQoL

Patient-reported data of HRQoL were registered using generic instruments, i.e., the medical outcome study (MOS) short form-36 (SF-36) questionnaire [19], the functional assessment of chronic illness therapy (FACIT)-Fatigue scale [20] and the 3-level EuroQoL research foundation 5-dimension (EQ-5D) health survey [21].

The MOS SF-36 is one of the most common generic questionnaires used for assessment of HRQoL in patients with different health conditions, as well as in the general population [19]. It contains 36 questions, analysis of which results in eight subscales representing different HRQoL aspects, i.e., physical functioning (PF), role physical (RP), bodily pain (BP), general health (GH), social functioning (SF), vitality (VT), role emotional (RE) and mental health (MH). The response from SF-36 was scored using the SF-36v2 manual [22], yielding subscale scores from 0 to 100. Next, the SF-36 subscales were computed according to a three-step procedure, including Z-score transformation and weighting based on the general US population to generate two summary measures, i.e., the physical component summary (PCS) and the mental component summary (MCS). Although all subscales are weighted in the derivation of both PCS and MCS, PF, RP, BP and GH are referred to as the physical aspects, and SF, VT, RE and MH are referred to as the mental aspects of SF-36. In terms of interpretation, high scores in SF-36 component summaries and subscales are considered a favourable perception of HRQoL and low scores are interpreted as poor HRQoL.

The FACIT-Fatigue scale is an instrument that includes 13 items and is designed to assess the level of fatigue over the preceding seven days. The scores generated have a span from 0 (maximal fatigue) to 52 (minimal fatigue), with scores < 30 representing severe fatigue.

The 3-level EQ-5D health survey consists of two distinct sections, i.e., a visual analogue scale (VAS), measuring patients’ health perception from 0 (worst health status) to 100 (best health status), and a descriptive system, consisting of a questionnaire that comprises five dimensions, i.e., self-care, mobility, usual activities, pain/discomfort and anxiety/depression. Each dimension is scored by the respondent, with possible answers being no problems (level 1), some/moderate problems (level 2), or extreme/major problems (level 3). Responses to these five questions are next summarised into a utility index score. In the present study, EQ-5D utility index scores were calculated based on the valuation of EQ-5D health states from a general US population sample [23]. In terms of interpretation, higher utility index scores represent a better HRQoL. “Full-health state” was defined as statement of no problems in all five dimensions [24].

Apart from numerical and statistical differences, we endorsed the concept of minimal clinically important differences (MCIDs), and registered fulfilment of MCIDs in comparisons between AMA users and non-users. Based on previous literature, we set the MCID for SF-36 PCS and MCS scores to ≥2.5 points and for SF-36 subscales to ≥5.0 points [25], for FACIT-Fatigue scores to ≥4 points [26], for EQ-5D utility index scores to ≥0.040 points, used for scores calculated using the US valuation algorithm [27], and for EQ-5D VAS scores to ≥10 points [28]. For MCIDs that in previous studies were meant for evaluation of changes of the HRQoL between different time points, with different benchmarks for improvement and worsening, we considered the greatest benchmark as the MCID for the respective HRQoL item in the cross-sectional design of the present study.

### 2.3. Evaluation of Disease Activity and Organ Damage

In the BLISS trials, SLE disease activity was measured using the SELENA-SLEDAI [18] and the classic British Isles Lupus Assessment Group Index (BILAG) [29] indices. Organ damage was assessed using the Systemic Lupus International Collaborating Clinics/American College of Rheumatology Damage Index (SDI) [30].

### 2.4. Patient Subgroups based on Organ-Specific Activity

For patient subgroup analyses, we evaluated associations between use of AMA and patient perceptions of HRQoL in study participants with active mucocutaneous, musculoskeletal and renal disease, herein defined as BILAG A or B in the respective domain.

### 2.5. Statistical Analysis

Data are presented as numbers (percentage) or means ± standard deviation (SD). Comparisons of continuous data between AMA users and non-users were conducted using the Mann-Whitney *U* test. The Pearson’s chi-square test was used to investigate contingent associations between binomial variables. Subsequently, linear regression analysis was carried out for comparisons yielding clinically important differences in order to adjust for potential confounding factors, selected based on previous literature [12,31,32,33]. Covariates included age, sex, ethnicity, SELENA-SLEDAI scores, SLE disease duration, SDI scores, prednisone (or equivalent) dose and use of immunosuppressants. Multivariable linear regression models included items that showed statistically significant associations in preceding univariable analysis. *p*-values < 0.05 were considered statistically significant. Statistical analyses were performed using the IBM SPSS software version 25 (IBM Corp., Armonk, NY, USA). GraphPad Prism 7 (GraphPad Software Inc., La Jolla, CA, USA) was used for the construction of graphs.

## 3. Results

### 3.1. Patient Characteristics

Of 1684 SLE patients recruited to the BLISS trials, 1098 patients were on AMA at the baseline evaluation (65.2%), and 94.1% were women. Demographics and SLE disease characteristics for the entire study population are presented in Table 1, and include AMA compounds and dose, as well as comparisons between AMA users and non-users. In Supplementary Materials Table S1, we present patient characteristics in the BLISS-52 and BLISS-76 trials separately.

Patients receiving AMA were younger than patients who were not on AMA, whereas no difference was seen in sex distributions. The groups had a similar composition of races/ethnic origins, with an overall greater representation of white/Caucasian patients, followed by indigenous American, Asian and black/African American patients. SELENA-SLEDAI scores did not differ between the AMA groups (9.6 ± 3.6 versus 10.0 ± 4.0; *p* = 0.145). AMA users (0.69 ± 1.15) had lower SDI scores compared with AMA non-users (0.95 ± 1.37; *p* < 0.001), and a shorter disease duration (6.1 ± 6.2 versus 7.0 ± 6.6 years; *p* = 0.007).

Fewer patients were on corticosteroids within the AMA group (84.6%) compared with AMA non-users (89.4%; *p* = 0.006), and AMA users had lower average prednisone equivalent doses (10.1 ± 8.5 versus 12.1 ± 8.8 mg/day; *p* < 0.001). Use of immunosuppressants was less frequent in AMA users (43.4%) compared with non-users (58.0%; *p* < 0.001).

### 3.2. MOS SF-36

**Figure 1:**
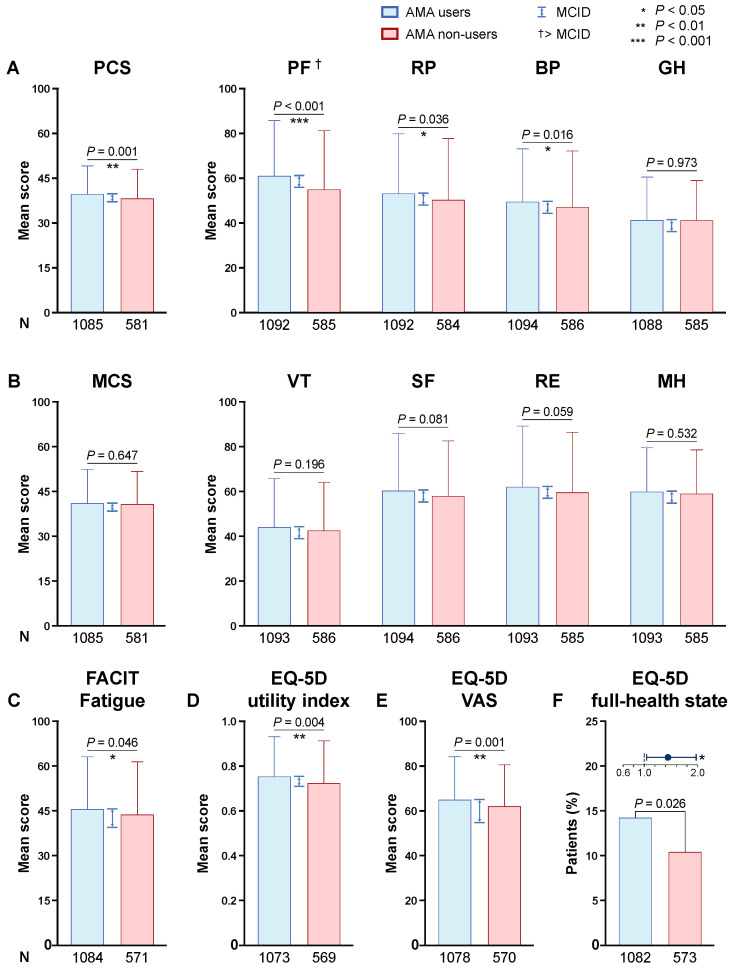
Comparisons of HRQoL between AMA users and non-users. This figure illustrates comparisons of HRQoL perceptions between patients with SLE who received AMA and patients with SLE who did not. Heights of the boxes represent mean HRQoL item scores (**A**–**E**) or percentage of patients (**F**), and whiskers indicate standard deviations. Vertical bidirectional arrows indicate MCIDs. The forest plot in panel F illustrates the odds ratio (circle) and 95% confidence interval (whiskers) of the corresponding comparison. Actual number of observations is indicated below the bars. Asterisks indicate statistically significant associations. AMA: antimalarial agents; SLE: systemic lupus erythematosus; HRQoL: health-related quality of life; FACIT-Fatigue: functional assessment of chronic illness therapy-Fatigue; EQ-5D: EuroQol research foundation 5-dimension; VAS: visual analogue scale; MCID: minimal clinically important difference.

As delineated in Figure 1, SLE patients who received AMA reported higher SF-36 PCS (39.6 ± 9.5 versus 38.1 ± 9.9; *p* = 0.001), physical functioning (61.1 ± 24.9 versus 55.0 ± 26.5; *p* < 0.001), role physical (53.2 ± 26.9 versus 50.3 ± 27.7; *p* = 0.036) and bodily pain (49.5 ± 23.8 versus 47.1 ± 25.3; *p* = 0.016) scores compared with patients who did not. Notably, only the difference in the physical functioning subscale was greater than the corresponding MCID. There were no differences between the AMA groups with regard to SF-36 MCS scores or SF-36 subscales scores representing the mental compartment. 

### 3.3. FACIT-Fatigue

Patients who received AMA (30.5 ± 11.8) reported better FACIT-Fatigue scores compared with patients who did not (29.3 ± 11.9; *p* = 0.046), yielding, however, no greater difference than the MCID (Figure 1).

### 3.4. EQ-5D

**Figure 2:**
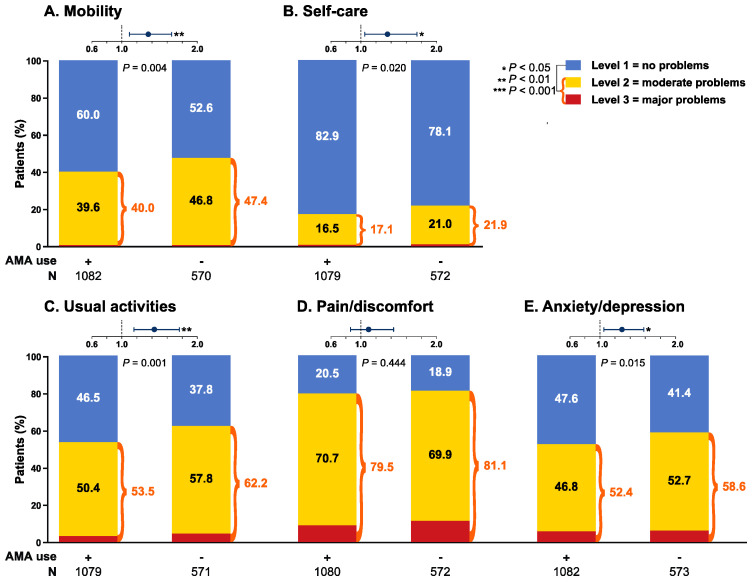
Response to EQ-5D dimensions in AMA users versus non-users. This figure illustrates comparisons between the response of patients with SLE who received AMA and the response of patients who did not receive AMA to the five different dimensions of the EQ-5D questionnaire, i.e., mobility (**A**), self-care (**B**), usual activities (**C**), pain/discomfort (**D**) and anxiety/depression (**E**). Proportions of patients reporting each one of the three levels (no problems, moderate problems, major problems) are indicated by colour-coded sections (blue, yellow, red) within the bars. *p*-values are derived from Pearson’s chi-square tests and signify comparisons of level 1 responders between AMA users and non-users. The forest plots illustrate the odds ratio (circles) and 95% confidence interval (whiskers) of the corresponding comparison. Actual number of observations is indicated below the bars. Asterisks indicate statistically significant associations. AMA: antimalarial agents.

Patients in the AMA group reported higher EQ-5D VAS scores (64.6 ± 19.4) compared with patients who did not receive AMA (61.7 ± 18.6; *p* < 0.001), but the difference was not clinically important (<MCID; Figure 1). Accordingly, AMA users reported better EQ-5D utility index scores (0.747 ± 0.185) than AMA non-users (0.720 ± 0.192; *p* = 0.004), but the difference did not reach the MCID. We next analysed the different dimensions of the questionnaire separately: we used the Pearson’s chi-square test to compare AMA groups in relation to patients reporting no problems versus moderate or major problems. In this analysis, a higher proportion of patients reporting no problems was seen among AMA users versus non-users with regard to mobility (60.0% versus 52.6%; odds ratio, OR: 1.35; 95% confidence interval, CI: 1.10–1.66; *p* = 0.004), self-care (82.9% versus 78.1%; OR: 1.35; 95% CI: 1.05–1.74; *p* = 0.020), usual activities (46.5% versus 37.8%; OR: 1.43; 95% CI: 1.16–1.76; *p* = 0.001) and anxiety/depression (47.6% versus 41.4%; OR: 1.29; 95% CI: 1.05–1.58; *p* = 0.015), but not pain/discomfort (20.5% versus 18.9%; OR: 1.11; 95% CI: 0.86–1.43; *p* = 0.444) (Figure 2). Finally, the proportion of patients experiencing “full-health state” was higher within AMA users (14.1% versus 10.3%; OR: 1.44; 95% CI: 1.04–1.97; *p* = 0.026; Figure 1).

### 3.5. Associations with SF-36 Physical Functioning

We next selected HRQoL aspects where the difference between AMA users and non-users exceeded the corresponding MCID, i.e., the SF-36 PF subscale, for further evaluation in relation to demographical and disease-associated factors with confounding potentiality, employing linear regression analysis.

**Figure 3:**
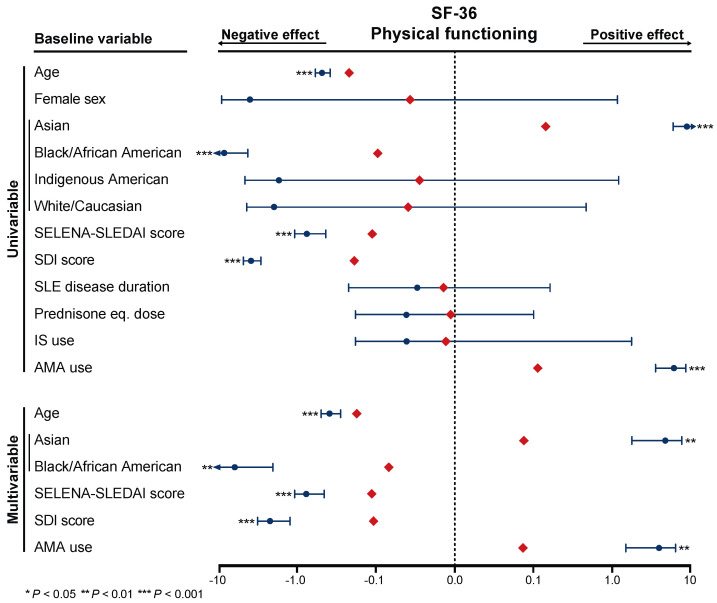
Association between AMA use and SF-36 physical functioning. The forest plots illustrate results from linear regression analysis, employed to investigate the association between AMA use (covariate) and SF-36 physical functioning (outcome), in relation to demographical and disease-specific factors. Factors showing statistically significant associations in univariable analysis were next included in a multivariable model. The dark blue circles represent the un-standardised coefficients, and the whiskers represent the 95% confidence intervals. The red diamonds represent the standardised coefficients. Asterisks indicate statistically significant associations. SF-36: short-form 36; PF: physical functioning; SLE: systemic lupus erythematosus; SELENA-SLEDAI: Safety of Estrogens in Lupus National Assessment SLE Disease Activity Index; SDI: Systemic Lupus International Collaborating Clinics/American College of Rheumatology Damage Index; IS: immunosuppressive; AMA: antimalarial agents.

In multivariable analysis, use of AMA was associated with higher SF-36 PF scores (standardised coefficient, β = 0.07; *p* = 0.002), independently of the other factors analysed (Figure 3). In the same model, Asian ancestry was also associated with a healthier perception of SF-36 PF (β = 0.08; *p* = 0.002), whereas African American origin (β = −0.07; *p* = 0.004), high SELENA-SLEDAI scores (β = −0.11; *p* < 0.001) and high SDI scores (β = −0.11; *p* < 0.001) were associated with lower SF-36 PF scores.

### 3.6. Stratification into Subgroups Based on Organ-Specific Activity

We next studied the impact of AMA use on HRQoL in SLE patients with active (BILAG A or B) mucocutaneous, musculoskeletal and renal disease. Similar to the findings in the total study population, patients with active mucocutaneous SLE who received AMA reported higher scores than patients who did not receive AMA in the SF-36 PCS and three of four physical subscales, i.e., PF, RP and BP, as well as SF from the mental compartment of SF-36; however, only the difference in the PF subscale was clinically important (Table 2). Among patients with active musculoskeletal manifestations, AMA users reported higher SF-36 PF scores (55.34 ± 24.10) than AMA non-users (49.98 ± 26.06; *p* = 0.002), and this difference exceeded the MCID (Table 3). Distributions of scores did not differ between the AMA groups in any other SF-36 subscale or component summaries (*p* = not significant, ns for all). Among patients with active renal SLE, AMA users reported higher scores than patients who did not receive AMA, exceeding the MCID in the SF-36 BP from the physical compartment, and the SF and RE subscales from the mental compartment, but none of these differences reached statistical significance (Table 4).

FACIT-Fatigue scores did not differ between AMA groups in any of the three subgroups studied. Among patients with mucocutaneous BILAG A or B, AMA users reported lower EQ-5D VAS (65.10 ± 19.27 versus 60.53 ± 18.99; *p* < 0.001) and utility index (0.747 ± 0.173 versus 0.716 ± 0.189; *p* = 0.003) scores compared with AMA non-users. These differences did not reach the level of MCID (Table 2). Among subjects with active musculoskeletal (Table 3) and renal (Table 4) disease, EQ-5D utility index scores did not differ between the AMA groups (*p* = ns for all), and the differences in EQ-5D VAS scores did not reach the level of MCID.

Notably, a higher proportion of AMA users versus non-users reported no problems within the mobility (60.2% versus 49.4%; OR: 1.55; 95% CI: 1.12–2.02; *p* = 0.001), self-care (82.9% versus 75.9%; OR: 1.54; 95% CI: 1.12–2.13; *p* = 0.009), usual activities (44.9% versus 34.3%; OR: 1.56; 95% CI: 1.19–2.05; *p* = 0.001) and anxiety/depression (47.1% versus 38.4%; OR: 1.43; 95% CI: 1.10–1.87; *p* = 0.009) EQ-5D dimensions among patients with active mucocutaneous disease, and within mobility (50.5% versus 43.9%; OR: 1.31; 95% CI: 1.01–1.69; *p* = 0.45) and self-care (78.2% versus 71.9%; OR: 1.40; 95% CI: 1.04–1.89; *p* = 0.026) among patients with active musculoskeletal disease. By contrast, in the active renal subgroup, proportions differed only in the anxiety/depression dimension (54.6% versus 34.8%; OR: 2.25; 95% CI: 1.20–4.24; *p* = 0.011).

## 4. Discussion

In the present post-hoc analysis of the BLISS-52 and BLISS-76 trials, we demonstrated that patients with active SLE receiving AMA reported better physical functioning than patients who were not on AMA. This association was clinically important, and independent of age, ethnic origin, disease activity and organ damage accrual. Furthermore, AMA users reported more favourable perceptions of mobility, ability to carry out self-care and usual activities, and level of anxiety or depression. Notably, a greater proportion of patients among AMA users experienced a full-health state, defined as no problems in all dimensions of the EQ-5D questionnaire.

In patients with SLE, AMA have been coupled with numerous beneficial effects, including reductions in disease activity, organ damage accrual and flare rates, as well as prolonged patient survival [8,9,10]. Mechanisms involved in the immunomodulatory effects of AMA include an altered peptide processing in antigen-presenting cells, reduced B cell activity and altered binding of anti-phospholipid antibody-β_2_-glycoprotein I complexes to phospholipid bilayers [34,35]. In the present study, we confirmed that SLE patients using AMA had accrued less organ damage and were on lower prednisone doses compared with patients not treated with AMA. Importantly, our study was not designed to address causality, and these observations could be explained, at least partly, by the fact that AMA users were younger and had a shorter SLE disease duration at the time of assessment. The rather low proportion of patients on AMA treatment (65%) signifies that the need for increased awareness of the favourable effects of AMA and for alignment with current recommendations [7] remains.

With regard to HRQoL, data on the impact of AMA have been scarce and conflicting. In one study comprising 277 SLE patients from Peru, past and current use of AMA was associated with a better perception of physical health, burden to others and body image [13], as assessed using the LupusQoL, an SLE-specific questionnaire for self-reported HRQoL [36]. In a Swedish cohort of 69 SLE patients with active disease, selected for treatment with biological agents, i.e., belimumab or rituximab, patients receiving AMA performed better in social functioning and mental health [12], based on self-reports using the SF-36 health survey [19]. By contrast, in a post-hoc analysis of the PLUS trial, a trial of hydroxychloroquine that comprised 166 SLE patients with low and stable disease activity, hydroxychloroquine concentrations were not associated with scores in any of the SF-36 component summaries or subscales, either at baseline or at month 7 [14]. Discrepancies in the different cohorts may be due to different disease phenotypes among the study participants. For example, patients selected for biological therapy in the aforementioned Swedish study had a more active disease, with an overrepresentation of active renal SLE, as opposed to the quiescent SLE cohort of the PLUS trial. Another explanation could be traced to the different instruments used to evaluate HRQoL. While generic indices provide information that has the advantage of being directly comparable with that of the general population and other disorders, disease-specific indices are expected to be more sensitive to change and perform better in discriminating across distinct subgroups of patients [37]. In this respect, the sole use of generic instruments in the present study may have contributed to omission of important disease-associated and disease-specific attributes that potentially influence the inventories and could be better captured by SLE-specific HRQoL questionnaires, such as the LupusQoL [36].

The BLISS populations included in this post-hoc analysis consisted of SLE patients with active disease despite standard of care treatment, with a high prevalence of mucocutaneous and musculoskeletal involvement. In the total study population, use of AMA was associated with better HRQoL perceptions in physical aspects of the SF-36, but the differences between AMA groups were moderate and only that in the physical functioning subscale exceeded the threshold of minimal clinically important difference. In the same fashion, the observed statistically significant difference in FACIT-Fatigue scores favouring the use of AMA was not clinically important. Lastly, differences in favour of AMA were also observed in the EQ-5D dimensions of mobility, self-care and usual activities. Although no MCIDs have to date been validated for the EQ-5D dimensions, patients using AMA had a 1.4-fold increased chance to report no problems in each one of the three aforementioned dimensions. It should be mentioned that factors such as concomitant medications, especially glucocorticoids, and SLE disease activity, as well as common comorbid conditions with immense impact on levels of pain and fatigue, such as fibromyalgia, may have influenced our findings. Comparisons yielding clinically important differences in HRQoL perception in AMA users versus non-users qualified for further exploration of independence and confounding potentiality. Importantly, glucocorticoid doses and use of immunosuppressants were not found to impact physical functioning, and the favourable impact of AMA use on physical functioning was independent of the negative impact of age, disease activity and organ damage in multivariable linear regression analysis.

Patients with SLE and mucocutaneous or musculoskeletal involvement suffer from a higher degree of HRQoL diminutions than SLE patients with other manifestations, especially regarding physical aspects [4]. When we herein analysed these subgroups of patients independently, AMA use was associated with more favourable perceptions in physical aspects of HRQoL, including SF-36 physical functioning and EQ-5D mobility, self-care and usual activities. Furthermore, patients with active mucocutaneous manifestations who received AMA also reported a better health profile in the anxiety or depression EQ-5D dimension compared with patients who did not. These findings further support current treatment recommendations, which advocate that patients with mucocutaneous and musculoskeletal involvement might particularly benefit from treatment with AMA. Currently, AMA remain the first-line systemic therapy for cutaneous SLE [7,38], and are considered an effective option for the management of lupus polyarthritis [35].

An observation of particular interest was that, in contrast to the entire study population and the subgroups of active mucocutaneous and musculoskeletal SLE, patients with active renal disease benefited from AMA regarding perceptions of mental HRQoL. These differences were found to be clinically important with respect to SF-36 social functioning and role emotional, and yielded a 2.3-fold increased chance to report no problems in the EQ-5D anxiety or depression dimension. These observations are in line with the aforementioned Swedish study of SLE patients, in which lupus nephritis was the most frequent clinical phenotype, showing that AMA users reported better scores than non-users in SF-36 social functioning and mental health [12]. The lack of statistical significance in some of the differences in mental HRQoL aspects in the present study may be due to the fact that patients with severe active lupus nephritis were, as per study protocol, excluded from the BLISS trials, resulting in a relatively low number of patients in the renal subgroup analysis, and, reasonably, a rather moderate renal activity in these patients. Further investigation of the impact of AMA on HRQoL in renal SLE is merited.

Despite the widely known benefits of AMA on SLE disease activity and course, non-adherence remains a major problem [14,39,40]. Costedoat-Chalumeau et al. found that the most common reasons for AMA treatment discontinuation by patient initiative included the perception of AMA not being an effective treatment and apprehension about potential side-effects [41]. This may be partially explained by the fact that the long-term benefits of AMA, which constitute a main reason for prescription, do not have a direct impact on SLE patients’ perception of health status. Indeed, while AMA prescription by physicians is predominantly steered by evidence with regard to both organ-specific and long-term benefits, the latter including atheroprotective effects and reduced flare rates [1,6], patients’ principal concerns have been shown to be related to their ability to perform physical and usual activities, as well as the degree of fatigue and pain [42]. In recent years, including patient-reported outcomes in shared therapeutic decision-making between physician and patient has received increasing embracement in all medical fields [43]. In this regard, our findings contribute with further evidence on the beneficial effects of AMA on SLE patients’ HRQoL and may provide support to motivate adherence to AMA therapy.

The cross-sectional design of our study constituted a major limitation. For example, it limited us from exploring a potential causality in the relationship between AMA use and HRQoL benefits. Furthermore, no quantification of blood AMA concentrations was attempted in the BLISS trials. Thus, medication non-adherence may have resulted in unintentional inaccuracies in our findings, which therefore have to be interpreted with caution. Information about comorbid conditions with confounding potentiality, such as fibromyalgia, was not available. Finally, patients with severe active lupus nephritis and neuropsychiatric SLE were excluded from the BLISS trials, and the conclusions of this study should therefore not be extrapolated to these patient subgroups. Nonetheless, the strengths of this investigation included the large study population, the diversity of patients enrolled from 32 different countries, the variety of instruments utilised to evaluate HRQoL and the extensive availability of homogeneous data in the BLISS-52 and BLISS-76 trials that allowed us to pool the two cohorts and adjust for multiple factors. To our knowledge, to date, this is the largest analysis of AMA use in relation to HRQoL in patients with SLE.

## 5. Conclusions

In the present cohort of 1684 patients with active SLE, mainly comprising patients with active mucocutaneous and musculoskeletal disease, we observed a clinically important benefit of AMA use with respect to physical functioning. Importantly, this effect was not impacted by patients’ age, ethnic origin, SLE disease activity or organ damage accrual. A particular benefit of AMA use on mental aspects of HRQoL was observed in the subgroup of patients with active renal disease. Results from this investigation provide further support in motivating adherence to AMA therapy. Exploration of a potential causality in the relationship between AMA use and favourable HRQoL in people living with SLE has merit.

## Figures and Tables

**Table 1:** Demographic characteristics and clinical data of antimalarial agents (AMA) users versus non-users.

Patient Characteristics	Pooled BLISS	AMA Use		*p* Value
		+	−	
Number of Patients	1684	1098	586	
Demographic Characteristics				
Age (years)	37.8 (11.5)	36.8 (11.4)	39.6 (11.5)	**<0.001**
Female sex	1585 (94.1%)	1033 (94.1%)	552 (94.2%)	0.922
Ethnicity				
Asian	353 (21.0%)	243 (22.1%)	110 (18.8%)	0.107
Black/African American	146 (8.7%)	98 (8.9%)	48 (8.2%)	0.610
Indigenous American	374 (22.2%)	254 (23.1%)	120 (20.5%)	0.212
White/Caucasian	798 (47.4%)	491 (44.7%)	307 (52.4%)	**0.003**
Clinical Data				
SELENA-SLEDAI score	9.7 (3.8)	9.6 (3.6)	10.0 (4.0)	0.145
SLE disease duration (years)	6.4 (6.3)	6.1 (6.2)	7.0 (6.6)	**0.007**
SDI score	0.78 (1.24)	0.69 (1.15)	0.95 (1.37)	**<0.001**
SDI score = 0	977 (58.1%)	673 (61.3%)	304 (52.0%)	**<0.001**
Glucocorticoid use	1453 (86.3%)	929 (84.6%)	524 (89.4%)	**0.006**
Prednisone eq. dose (mg/day)	10.8 (8.7)	10.1 (8.5)	12.1 (8.8)	**<0.001**
AMA use	1098 (64.8%)	1098 (100%)	N/A	N/A
Hydroxychloroquine	836 (49.6%)	836 (76.1%)	N/A	N/A
Chloroquine	265 (15.7%)	265 (24.1%)	N/A	N/A
Other antimalarial agents *	5 (0.3%)	5 (0.5%)	N/A	N/A
Hydroxychloroquine eq. dose (mg/day)	219.6 (183.5)	336.2 (111.0)	N/A	N/A
Immunosuppressants	816 (48.5%)	476 (43.4%)	340 (58.0%)	**<0.001**
Azathioprine	389 (23.1%)	221 (20.1%)	168 (28.7%)	**<0.001**
Methotrexate	231 (13.7%)	144 (13.1%)	87 (14.8%)	0.325
Mycophenolic acid	189 (11.2%)	104 (9.5%)	85 (14.5%)	**0.002**

Data are presented as numbers (percentage) or means (standard deviation). Statistically significant *p*-values are indicated in bold. * Mepacrine, mepacrine hydrochloride, quinine sulphate. AMA: antimalarial agents; SELENA-SLEDAI: Safety of Estrogens in Lupus National Assessment Systemic Lupus Erythematosus Disease Activity Index; SDI: Systemic Lupus International Collaborating Clinics (SLICC)/American College of Rheumatology (ACR) Damage Index. N/A: not applicable.

**Table 2:** Comparisons of HRQoL between AMA users and non-users in patients with active mucocutaneous disease.

HRQoL Items		AMA Users		AMA Non-Users		*p* Value	MCID
Number of Patients		638		353			
SF-36							
Physical component summary		39.63	(9.31)	37.82	(9.76)	**0.006**	No
Mental component summary		40.60	(11.44)	40.02	(10.82)	0.448	No
Physical functioning		61.46	(24.71)	54.16	(26.58)	**<0.001**	**Yes**
Role physical		52.51	(26.59)	49.00	(27.52)	**0.033**	No
Bodily pain		48.81	(22.71)	46.17	(24.82)	**0.038**	No
General health		41.40	(19.22)	40.98	(18.09)	0.763	No
Vitality		43.86	(21.34)	41.91	(21.33)	0.175	No
Social functioning		60.63	(25.14)	57.01	(25.01)	**0.043**	No
Role emotional		61.13	(27.44)	58.96	(27.07)	0.165	No
Mental health		59.20	(20.19)	57.60	(19.31)	0.245	No
FACIT-Fatigue							
Score		30.32	(11.74)	28.87	(12.09)	0.077	No
EQ-5D							
Utility index		0.747	(0.173)	0.716	(0.189)	**0.003**	No
VAS		65.10	(19.27)	60.53	(18.99)	**<0.001**	No
EQ-5D Dimensions							
Mobility	L 1	377	(60.2%)	169	(49.4%)	**0.001**	N/A
	L 2	249	(39.8%)	172	(50.3%)		
	L 3	0	(0%)	1	(0.3%)		
Self-care	L 1	518	(82.9%)	261	(75.9%)	**0.009**	N/A
	L 2	103	(16.5%)	78	(22.7%)		
	L 3	4	(0.6%)	5	(1.5%)		
Usual activities	L 1	281	(44.9%)	118	(34.3%)	**0.001**	N/A
	L 2	331	(52.9%)	213	(61.9%)		
	L 3	14	(2.2%)	13	(3.8%)		
Pain or discomfort	L 1	116	(18.6%)	58	(16.9%)	0.510	N/A
	L 2	460	(73.6%)	248	(72.1%)		
	L 3	49	(7.8%)	38	(11.0%)		
Anxiety or depression	L 1	295	(47.1%)	132	(38.4%)	**0.009**	N/A
	L 2	295	(47.1%)	196	(57.0%)		
	L 3	36	(5.8%)	16	(4.7%)		
Full-health state		77	(12.3%)	34	(9.9%)	0.262	N/A

Data are presented as means (standard deviation (SD)) or numbers (percentage). In comparisons of SF-36, FACIT-Fatigue, EQ-5D utility index and EQ-5D VAS scores, *p*-values are derived from Mann-Whitney *U* tests. In comparisons of EQ-5D dimensions, *p*-values are derived from Pearson’s chi-square tests and signify comparisons between AMA groups in relation to patients reporting no problems (level 1) versus moderate or major problems (level 2 and level 3 combined). Statistically significant *p*-values and differences exceeding the MCID are indicated in bold. AMA: antimalarial agents; MCID: minimal clinically important difference; SF-36: Short Form-36; FACIT: Functional Assessment of Chronic Illness Therapy; EQ-5D: EuroQol 5 Dimensions; VAS: visual analogue scale; L: level; N/A: not applicable.

**Table 3:** Comparisons of HRQoL between AMA users and non-users in patients with active musculoskeletal disease.

HRQoL Items		AMA Users		AMA Non-Users		*p* Value	MCID
Number of Patients		363		372			
SF-36							
Physical component summary		37.18	(9.12)	35.95	(8.99)	**0.042**	No
Mental component summary		40.22	(11.39)	40.08	(11.58)	0.903	No
Physical functioning		55.34	(24.10)	49.98	(26.06)	**0.002**	**Yes**
Role physical		48.87	(25.78)	46.27	(25.87)	0.131	No
Bodily pain		43.26	(21.24)	40.96	(22.31)	0.053	No
General health		38.89	(18.83)	39.94	(17.45)	0.298	No
Vitality		40.16	(21.53)	39.45	(21.05)	0.584	No
Social functioning		56.91	(25.85)	55.01	(24.47)	0.361	No
Role emotional		59.33	(27.46)	57.40	(27.77)	0.207	No
Mental health		58.54	(19.68)	57.80	(20.50)	0.654	No
FACIT-Fatigue							
Score		28.37	(11.93)	27.22	(11.96)	0.167	No
EQ-5D							
Utility index		0.706	(0.182)	0.684	(0.195)	0.080	No
VAS		61.76	(19.91)	59.21	(18.28)	**0.042**	No
EQ-5D Dimensions							
Mobility	L 1	318	(50.5%)	157	(43.9%)	**0.045**	N/A
	L 2	311	(49.4%)	200	(55.9%)		
	L 3	1	(0.2%)	1	(0.3%)		
Self-care	L 1	491	(78.2%)	258	(71.9%)	**0.026**	N/A
	L 2	132	(21.0%)	98	(27.3%)		
	L 3	5	(0.8%)	3	(0.8%)		
Usual activities	L 1	230	(36.7%)	111	(31.0%)	0.072	N/A
	L 2	374	(59.6%)	224	(62.6%)		
	L 3	23	(3.7%)	23	(6.4%)		
Pain or discomfort	L 1	66	(10.5%)	31	(8.6%)	0.345	N/A
	L 2	489	(77.7%)	276	(76.9%)		
	L 3	74	(11.8%)	52	(14.5%)		
Anxiety or depression	L 1	261	(41.4%)	147	(40.8%)	0.855	N/A
	L 2	326	(51.7%)	188	(52.2%)		
	L 3	43	(6.8%)	25	(6.9%)		
Full-health state		43	(6.8%)	20	(5.6%)	0.431	N/A

Data are presented as means (SD) or numbers (percentage). In comparisons of SF-36, FACIT-Fatigue, EQ-5D utility index and EQ-5D VAS scores, *p*-values are derived from Mann-Whitney *U* tests. In comparisons of EQ-5D dimensions, *p*-values are derived from Pearson’s chi-square tests and signify comparisons between AMA groups in relation to patients reporting no problems (level 1) versus moderate or major problems (level 2 and level 3 combined). Statistically significant *p*-values and differences exceeding the MCID are indicated in bold. AMA: antimalarial agents; MCID: minimal clinically important difference; SF-36: Short Form-36; FACIT: Functional Assessment of Chronic Illness Therapy; EQ-5D: EuroQol 5 Dimensions; VAS: visual analogue scale; L: level; N/A: not applicable.

**Table 4:** Comparisons of HRQoL between AMA users and non-users in patients with active renal disease.

HRQoL Items		AMA Users		AMA Non-Users		*p* Value	MCID
Number of Patients		112		67			
SF-36							
Physical component summary		40.70	(9.88)	39.39	(11.87)	0.471	No
Mental component summary		41.44	(10.89)	39.54	(11.42)	0.288	No
Physical functioning		60.69	(26.23)	57.93	(28.50)	0.547	No
Role physical		56.10	(28.05)	52.33	(31.74)	0.503	No
Bodily pain		54.42	(26.78)	49.28	(31.18)	0.149	**Yes**
General health		43.19	(20.16)	40.34	(19.56)	0.309	No
Vitality		46.27	(22.28)	44.40	(24.19)	0.516	No
Social functioning		62.39	(25.14)	56.90	(26.68)	0.138	**Yes**
Role emotional		63.58	(25.69)	57.90	(27.58)	0.183	**Yes**
Mental health		59.99	(19.73)	57.87	(19.89)	0.545	No
FACIT-Fatigue							
Score		32.19	(11.64)	30.03	(13.11)	0.357	No
EQ-5D							
Utility index		0.768	(0.206)	0.733	(0.221)	0.286	No
VAS		65.61	(21.59)	59.86	(19.48)	**0.041**	No
EQ-5D Dimensions							
Mobility	L 1	69	(63.9%)	35	(53.0%)	0.156	N/A
	L 2	39	(36.1%)	31	(47.0%)		
	L 3	0	(0%)	0	(0%)		
Self-care	L 1	84	(78.5%)	54	(81.8%)	0.598	N/A
	L 2	22	(20.6%)	12	(18.2%)		
	L 3	1	(0.9%)	0	(0%)		
Usual activities	L 1	52	(48.6%)	30	(45.5%)	0.688	N/A
	L 2	54	(50.5%)	32	(48.5%)		
	L 3	1	(0.9%)	4	(6.1%)		
Pain or discomfort	L 1	33	(30.8%)	22	(33.3%)	0.732	N/A
	L 2	63	(58.9%)	35	(53.0%)		
	L 3	11	(10.3%)	9	(13.6%)		
Anxiety or depression	L 1	59	(54.6%)	23	(34.8%)	**0.011**	N/A
	L 2	45	(41.7%)	37	(56.1%)		
	L 3	4	(3.7%)	6	(9.1%)		
Full-health state		27	(25.0%)	13	(19.7%)	0.420	N/A

Data are presented as means (SD) or numbers (percentage). In comparisons of SF-36, FACIT-Fatigue, EQ-5D utility index and EQ-5D VAS scores, *p*-values are derived from Mann-Whitney *U* tests. In comparisons of EQ-5D dimensions, *p*-values are derived from Pearson’s chi-square tests and signify comparisons between AMA groups in relation to patients reporting no problems (level 1) versus moderate or major problems (level 2 and level 3 combined). Statistically significant *p*-values and differences exceeding the MCID are indicated in bold. AMA: antimalarial agents; MCID: minimal clinically important difference; SF-36: Short Form-36; FACIT: Functional Assessment of Chronic Illness Therapy; EQ-5D: EuroQol 5 Dimensions; VAS: visual analogue scale; L: level; N/A: not applicable.

## Supplementary Materials

The following are available online at https://www.mdpi.com/2077-0383/9/6/1813/s1, Table S1: Characteristics of AMA users versus non-users in BLISS-52 and BLISS-76.
