# Supplementary material for: Use of Antimalarial Agents Is Associated with Favourable Physical Functioning in Patients with Systemic Lupus Erythematosus
Source: J Clin Med. 2020 Jun 10;9(6):1813. doi: 10.3390/jcm9061813 (PMC7355692; doi:10.3390/jcm9061813)
Supplement: Supplementary file 1 [file jcm-09-01813-s001.pdf]

Supplementary Table S1. Characteristics of AMA users versus non-users in BLISS-52 and BLISS-76.

Study	BLISS-52			BLISS-76		
**AMA**	**Users**	**Non-Users**	***p* Value**	**Users**	**Non-Users**	***p* Value**
Number of patients	579	286		519	300	
Demographics						
Age (years)	34.9 (11.0)	36.8 (11.1)	**0.009**	39.0 (11.4)	42.2 (11.3)	**<0.001**
Female sex	546 (94.3%)	275 (96.2%)	0.243	487 (93.8%)	277 (92.3%)	0.408
Ethnicity						
Asian	226 (39.0%)	101 (35.3%)	0.289	17 (3.3%)	9 (3.0%)	0.828
Black/African American	20 (3.5%)	10 (3.5%)	0.974	78 (15.0%)	38 (12.7%)	0.350
Indigenous American	189 (32.6%)	85 (29.7%)	0.385	65 (12.5%)	35 (11.7%)	0.718
White/Caucasian	139 (24.0%)	90 (31.5%)	**0.019**	352 (67.8%)	217 (72.3%)	0.177
Clinical data						
SELENA-SLEDAI score	9.7 (3.6)	9.9 (4.1)	0.755	9.5 (9.0)	10.0 (3.8)	0.067
SLE disease duration (years)	5.1 (5.1)	5.8 (5.7)	0.130	7.2 (7.1)	8.1 (7.2)	**0.045**
SDI score	0.48 (0.90)	0.75 (1.15)	**<0.001**	0.92 (1.33)	1.13 (1.54)	0.054
SDI score = 0	400 (69.1%)	165 (57.7%)	**0.001**	273 (52.6%)	139 (46.5%)	0.092
Glucocorticoid use	555 (95.9%)	275 (96.2%)	0.834	374 (72.1%)	249 (83.0%)	**<0.001**
Prednisone dose (mg/day)	11.9 (8.5)	14.3 (9.0)	**<0.001**	8.1 (8.1)	10.0 (8.2)	**<0.001**
Immunosuppressants	219 (37.8%)	142 (49.7%)	**0.001**	257 (49.5%)	198 (66.0%)	**<0.001**
Azathioprine	134 (23.1%)	88 (30.8%)	**0.016**	87 (16.8%)	80 (26.7%)	**0.001**
Methotrexate	46 (7.9%)	33 (11.5%)	0.084	98 (18.9%)	54 (18.0%)	0.754
Mycophenolic acid	31 (5.4%)	21 (7.3%)	0.247	73 (14.1%)	64 (21.3%)	**0.007**

Data are presented as numbers (percentage) or means (standard deviation). Statistically significant *p*-values are indicated in bold. AMA: antimalarial agents; SELENA-SLEDAI: Safety of Estrogens in Lupus National Assessment Systemic Lupus Erythematosus Disease Activity Index; SDI: Systemic Lupus International Collaborating Clinics (SLICC)/American College of Rheumatology (ACR) Damage Index.
